# Screening and selection of peptides specific for esophageal cancer cells from a phage display peptide library

**DOI:** 10.1186/1749-8090-9-76

**Published:** 2014-04-29

**Authors:** Zhe-Feng Zhang, Xue Shan, Yong-Xin Wang, Wei Wang, Shi-Yun Feng, You-Bin Cui

**Affiliations:** 1 Department of Thoracic Surgery, The First Hospital of Jilin University, 71 Xinmin Street, 130021 Changchun, China; 2 Department of Cardiac Surgery, The First Hospital of Jilin University, Changchun, China; 3 Department of Gynaecology and Obstetrics, The First Hospital of Jilin University, Changchun, China

**Keywords:** Phage display, Esophageal cancer cells, Subtractive screening, Targeted cancer therapy

## Abstract

**Background:**

Esophageal cancer is a common malignant tumor of the gastrointestinal tract and is typically diagnosed at an advanced stage due to the absence of early clinical symptoms. Although surgery, chemotherapy, and radiotherapy represent the major treatment methods employed for this cancer, the prognosis of esophageal cancer remains poor.

**Methods:**

A Ph.D.-12^TM^ Phage Display Peptide Library was screened using an esophageal cancer cell line, Eca109, and a normal esophageal epithelial cell line to identify novel ligands that selectively bind the surface of esophageal cancer cells with high affinity.

**Results:**

Two polypeptides were isolated that exhibited higher binding affinities and specificity for the Eca109 cells. These peptides were further validated using enzyme-linked immunosorbent assays (ELISAs), immunofluorescence assays, and immunohistochemistry assays.

**Conclusion:**

Two polypeptides with high binding affinities to esophageal cancer cells were isolated from the Ph.D.-12^TM^ Phage Display Peptide Library. Further studies are needed to characterize the biological effects of these polypeptides and to explore the potential for these peptides to be used for the early screening of esophageal cancer or for cell-targeted therapies that would reduce the toxic side effects of cancer treatment.

## Introduction

Esophageal cancer is a common gastrointestinal malignancy that has a 5-year survival rate of 10% [1]. Although treatment strategies include combinations of chemotherapy, radiation, and surgery, the rates of tumor recurrence and metastasis for this disease remain high [2]. In addition, esophageal cancer is usually diagnosed in its advanced stages due to an absence of obvious symptoms in its early stages. As a result, surgery is not always a treatment option [3].

In 1985, it was first reported that exogenous DNA could be integrated into the genes that encode surface proteins of filamentous phage [4]. As a result, “fusion phage” could be created, and these phage could be enriched for those with high affinity for a particular sequence. It was subsequently demonstrated that this model could provide a simple way of screening specific polypeptides or proteins against a target gene [5]. Tumor cells often express cell surface antigens that are tumor-associated or tumor-specific. Consequently, peptide phage display technology can select for peptide ligands that have high specificity and affinity for cell surface proteins in both *in vitro* and *in vivo* models. Indeed, studies have identified peptide ligands which are useful for tumor-targeted diagnosis and treatment [6-8]. For example, peptides that specifically bind HepG2 human liver cancer cells were identified using subtractive screening of a seven peptide phage display library [9]. In addition, screening of phage display libraries has been used to isolate peptides that specifically bind tumor blood vessels *in vivo*[10]. These screened peptides were then coupled to the anticancer drug, doxorubicin, in order to enhance the efficacy, and reduce the toxicity, of this drug against human breast cancer xenografts in nude mice [11]. However, the use of phage display to identify sequences which bind human esophageal cancer cells has not previously been reported.

In recent years, phage display technology has been used to identify ligands and short peptides with binding activity to a variety of molecules on the surface of tumor cells [12,13]. By providing a direct link between an experimental phenotype and an encapsulated genotype, cell-specific peptides can be screened to identify those with high affinity and specificity for a given target [14]. These ligands can then be used to identify new targets for cancer therapies or to facilitate drug design [15,16]. For example, the conjugation of phage-encoded short peptides with liposomes or nanochemotherapy drugs has achieved higher concentrations of drugs in targeted tumor tissues, thereby reducing the side effects experienced by normal tissues [17].

In this study, the esophageal cancer cell line, Eca109, normal human esophageal epithelial cells, and a phage peptide library were used to perform three rounds of subtractive screening to identify phage that specifically bind Eca109 cells. The phage clones isolated were subsequently characterized by enzyme-linked immunosorbent assays (ELISAs), immunofluorescence, and immunohistochemical assays.

## Materials and methods

### Cells and reagents

The Ph.D.-12^TM^ Phage Display Peptide Library and *E. coli* ER2738 were purchased from New England Biolabs (Beverly, MA, USA). The Eca109 cell line and human esophageal cancer tissue sections were obtained from the tissue library of the First Hospital of Jilin University (Changchun, China). Anti-M13 mouse monoclonal antibody was purchased from Amersham Biosciences (Piscataway, NJ, USA). Horseradish peroxidase (HRP)-labeled goat anti-mouse antibody, fluorescein isothiocyanate (FITC)-labeled goat anti-mouse antibody, proteose peptone, and yeast extract were purchased from Shanghai Rui Qi Biological Technology Co., Ltd (Shanghai, China).

### Cell culture and culture of E. coli ER2738

Eca109 cells were maintained in RPMI1640 culture medium containing 10% fetal bovine serum in an incubator at 37°C and 5% CO_2_. Cells were passaged at 80% confluence. Normal human primary esophageal epithelium cells were obtained and incubated in D-Hanks solution for 20 min. The cells were then digested with collagenase to obtain epithelial cells which were subsequently maintained in RPMI1640 medium. *E. coli* ER2738 were plated on Luria-Bertani-tetracycline (LB-Tet) plates, were incubated at 37°C overnight, and then were inoculated into LB medium to achieve log-phase growth.

### Amplification of phage and subtractive screening of a phage display peptide library (subtraction biopanning)

Phage expressing the Ph.D.-12^TM^ Phage Display Peptide Library were added to LB medium containing *E. coli* ER2738 in log-phase growth, and Eca109 cells and normal human esophageal epithelial cells were digested with 0.25% trypsin. Phage (10 μl) were then added to 5 × 10^6^ Eca109 cells and 5 × 10^6^ normal human esophageal epithelial cells to perform a round of subtractive screening. A total of three rounds of subtractive screening were performed, and *E. coli* ER2738 were infected with the phage recovered from each round of screening. For the second and third rounds of selection, the volume of added phage remained the same, yet the incubation time with the Eca109 cells decreased to 45 min and 30 min, respectively. In contrast, the incubation time with the normal esophageal cells increased to 1.25 h and 1.5 h, respectively. In addition, Tris-buffered saline/Tween-20 (TBST) that was used for the wash steps following the second and third rounds of selection was increased from 0.3% (v/v) to 0.5% (v/v), respectively.

### Selection and amplification of positive clones

After three rounds of subtractive screening, 20 positive clones (represented as blue plaques on selection plates) were randomly selected. These selected plaques were propagated in *E. coli* ER2738 and stored in 60% sterilized glycerol at 20°C.

### Positive phage clones were confirmed using enzyme-linked immunosorbent assays (ELISAs)

Eca109 cells and normal esophageal cells were plated in 96-well plates 24 h prior to the addition of positive phage clones. After 1 h at 37°C, the wells were washed to remove unbound phage. Anti-M13 monoclonal antibody (1:5000) was then added to each well to detect the presence of M13 coat protein pIII, and therefore, the remaining bound phage. Wells containing each cell type alone were used as blank controls. After 2 h, the plates were washed, and HRP-labeled goat anti-mouse antibody (1:5000) was added. After 2 h, the plates were washed again and 3,3′,5,5′-tetramethylbenzidine (TMB) substrate was added to each well. Absorbance values at 490 nm were subsequently recorded using a plate reader.

### Immunofluorescence and immunohistochemical assays

Eca109 cells and normal esophageal epithelial cells were resuspended in RPMI1640 containing 10% fetal bovine serum and seeded in 24-well plates. Upon reaching confluence, cells were fixed with 4% paraformaldehyde and then selected phage were added. After an incubation at 37°C and 5% CO_2_ for 2 h, anti-M13 monoclonal antibody and FITC-labeled goat anti-mouse antibody were sequentially added to each well, with 1 h incubations completed for each. Images were subsequently acquired using fluorescence microscopy.

Positive phage clones were also added to both normal and cancerous esophageal tissue sections. After 30 min, these tissues were sequentially incubated with primary and biotin-conjugated secondary antibodies for 1 h each. HRP-labeled streptavidin was subsequently applied, followed by 3,3′-diaminobenzidine tetrahydrochloride (DAB) reagent. Images were acquired using a light microscope.

### Sequencing of the positive clones

A polyethylene glycol (PEG)/NaCl solution was added to amplified phage before a buffer solution of sodium iodide and ethanol were added. After incubating samples at 37°C for 10 min, precipitated phage were resuspended in Tris-EDTA (TE) buffer. The sequencing primer, 5′-CCCTCATAGTTAGCGTAACG-3′ (96 g III), was used for automated DNA sequencing. Homology analyses were performed using the Amersham Biosciences database to deduce the amino acid sequences of the positive clones isolated.

## Results

### Phage enrichment after subtractive screening

**Figure 1:**
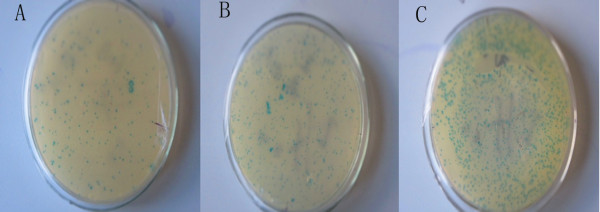
**Enrichment of phage achieved with each round of selection from a Ph.** D.-12TM phage display peptide library using Eca109 cells. **(A-C)** Dilution of phage at 1:100 were plated for rounds 1 and 2, then a dilution of 1:1000 was plated for round 3.

Three rounds of subtractive screening of a phage peptide library were performed using Eca109 as the target cells and normal human esophageal epithelial cells as the absorber cells. Phage titers were determined after each round of screening (Figure 1), and the recovery rates were found to increase 103-fold after three rounds of selection (Table 1). Thus, binding of phage to Eca109 cells was enriched.

**Table 1:** Enrichment of positive phage clones by subtraction biopanning

**Rounds**	**Input phage (pfu)**	**Output phage (pfu)**	**Recovery**
1	2.0 × 10^11^	4.2 × 10^3^	2.1 × 10^-8^
2	2.0 × 10^11^	2.5 × 10^5^	1.25 × 10^-6^
3	2.0 × 10^11^	6.8 × 10^6^	3.4 × 10^-5^

Pfu: plaque forming unit.

### Binding assays of the positive phage clones

**Figure 2:**
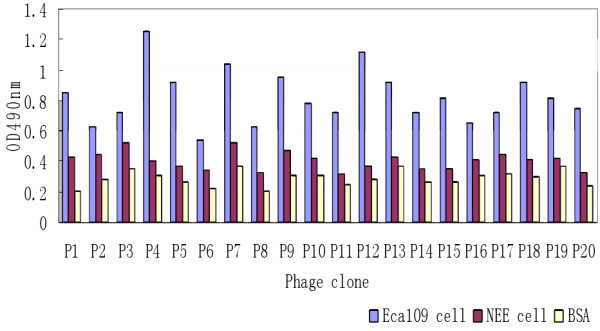
**An ELISA was performed to detect binding of phage clones to Eca109 cells (blue), normal human esophageal epithelial cells (NEE; red), and bovine serum albumin cells (BSA; yellow).** Data are presented as the mean ± SD.

For the phage obtained following the third round of screening, binding assays were performed using ELISAs. For phage clones: P1, P4, P5, P8, P9, P12, P15, P18, and P20, the OD_490nm_ ratio for the binding of these peptides to Eca109 cells versus a blank control was greater than three, indicating that these nine clones specifically bound Eca109 cells (Figure 2). These nine clones were further characterized in ELISAs containing Eca109 cells and normal esophageal cells. Of the nine clones, only clones P4 and P12 were found to selectively bind the Eca109 cells.

### Immunohistochemical characterization

**Figure 3:**
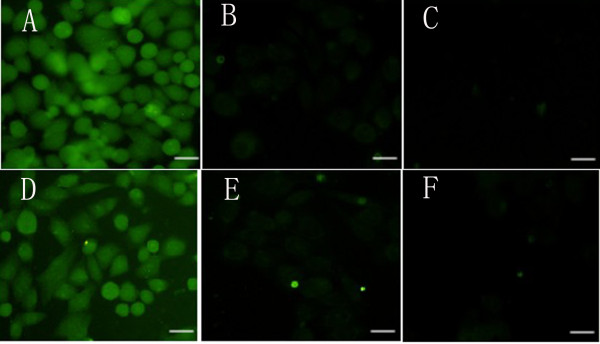
**Immunofluorescen labeled phage clones binding Eca109 cells and normal human esophageal epithelial cells (magnification, 400×).** Two clones, P4 and P12, were evaluated for their binding to Eca109 cells **(A, D)**, to normal esophageal epithelial cells **(B, E)**, and to controls (e.g., cells not incubated with anti-M13 antibody) **(C, F)**, respectively.

After phage clones, P4 and P12, were incubated with Eca109 cells, bound phage were subsequently detected using fluorescently labeled antibodies. A fluorescent signal was observed at the cell surface of both Eca109 cell samples (Figure 3, A & D). In contrast, a weak fluorescent signal was observed for normal esophageal cells incubated with the same two phage clones (Figure 3, B & E).

**Figure 4:**
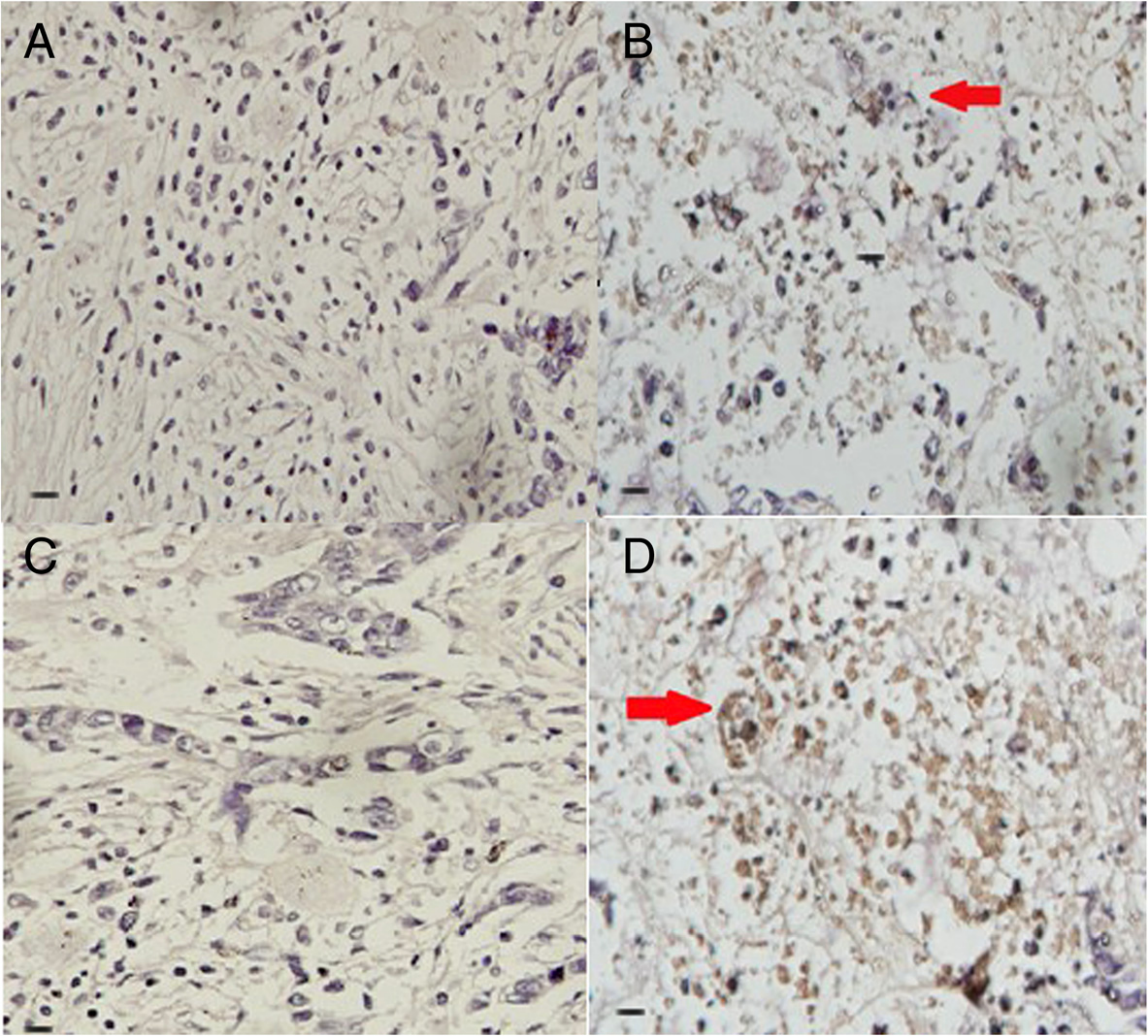
**DAB staining of P4 and P12 phage clones incubated with different tissues.** Esophageal cancer tissues **(A, C)** and normal esophageal tissues **(B, D)** were incubated, respectively (magnification, 400×).

Similar results were obtained in DAB immunochemistry assays. For example, positive staining (e.g., a brown color) was associated with esophageal cancer cells incubated with phage clones P4 and P12 (Figure 4, A & C), and was not observed for normal esophageal tissues incubated with the same two clones (Figure 4, B & D).

### Sequencing of positive clones

**Figure 5:**
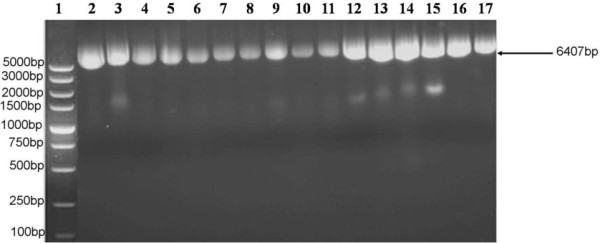
**Agarose gel electrophoresis of DNA obtained from eight colonies of P4 and P12 phage clones, in lanes 2–9 and 10–17, respectively.** The expected band is 6407 bp, as indicated with an arrow.

**Figure 6:**
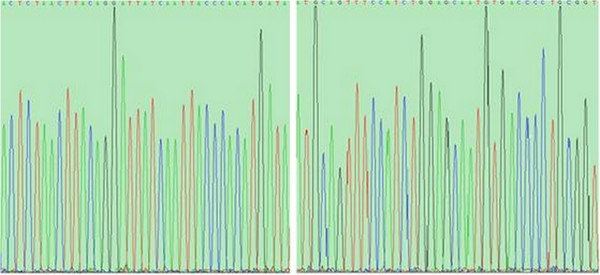
**DNA sequencing chromatogram for the P4 and P12 phage clones.** Two clones, P4 **(A)** and P12 **(B)** were evaluated, respectively.

Prior to sequencing, phage clones P4 and P12 were analyzed by agarose gel electrophoresis (Figure 5). An approximately 6.4 kb band was visualized for eight clones of P4 and eight clones of P12. When the aP4 and P12 clone were subsequently sequenced (Figure 6), the amino acid sequences identified were: Arg-Ala-Leu-Ala-His-Pro-Arg-Asp-His-Pro-Asp-Leu (R-A-L-A-H-P-R-D-H-P-D-L) and Ala-Thr-Cys-Ser-Met-Leu-Leu-Ser-Arg-Asn-Glu-Ala (A-T-C-S-M-L-L-S-R-N-E-A), respectively. There was no homology observed between the two sequences (Table 2). In addition, no homology between these two sequences and the protein sequences of the Amersham Biosciences database were observed.

**Table 2:** DNA and protein sequences for two positively selected phage clones

**Clones**	**DNA sequence**	**Peptide sequence**
P4	ACTCTAACTTACAGGATTACAATTACCCACATGATA	RALAHPRDHPDL
P12	ATGCAGTTTCCATCTGGAGCAATGTGACCCTGCGGT	ATCSMLLSRNEA

## Discussion

Solid phase-, liquid-, and whole cell-based screening methods are most commonly used for phage display selections. However, direct screening of antibody libraries against intact cells allows specific antibodies to be selected under normal physiological conditions [18]. Accordingly, a whole-cell screening method was used in the present study, with Eca109 cells used as target cells, normal human esophageal cells used as absorber cells, and a 12 peptide phage library used as a source of randomized peptides. While ELISA, immunofluorescence and immunohistochemistry assays were used to validate the binding affinities of two positive phage clones, sequencing of the positive clones did not find any homology between the sequences obtained and a protein database. This may be due to the diversity of surface receptors present on tumor cells, or a mutation that developed in the peptide sequences during screening. Consequently, further studies are needed to characterize the biological characteristics of these peptides.

Due to the complex surface of esophageal cancer cells, false positive results may inevitably occur, even when subtractive screening and strict screening criteria are applied for phage selection [19]. In addition, abnormal expression of surface molecules may occur during the *in vitro* culturing of Eca109 cells. However, selective binding of the isolated peptides to esophageal cancer cells versus normal esophageal cells indicates that novel tumor-specific antigens are present on the surface of esophageal cells, or that these antigens are present at low levels on the cell surface of normal cells. Either condition provides valuable insight into the development of molecular markers that could be applied to the diagnosis of esophageal cancer in its early stages, and/or the development of novel drug targets. Since expression of the selected clones was only characterized using Eca109 esophageal cancer cells, further studies are needed to evaluate the expression of the selected clones using human esophageal cancer samples. This may provide additional support for the use of these peptides in developing tumor biomarker assays. In addition, functional studies (particularly loss-of-function studies) may further elucidate the actions of the selected clones during esophageal carcinoma tumorigenesis. More comprehensive studies are also needed to determine whether the identified peptides may be applied to the diagnosis of esophageal cancer [20]. While the use of monoclonal antibodies for cancer therapy has been extensively characterized and developed [21,22], peptide ligands as targeted molecular therapies provide additional advantages such as: rapid penetration of tissues, rapid clearance from the blood, they are easily incorporated into delivery vectors, and they have exhibited low immunogenicity.

In summary, two phage clones exhibited specific binding and high affinity for human esophageal cancer cells compared with normal human esophageal cells. Further studies are needed to determine whether these peptides may be applied to the early diagnosis of esophageal cancer, or have the potential to serve as therapeutic targets.

## Conclusion

Two phage clones that were isolated from the Ph.D.-12TM Phage Display Peptide Library exhibited more specific binding and higher affinity for human esophageal cancer cells compared with normal human esophageal cells. These peptides were further validated using ELISAs, immunofluorescence assays, and immunohistochemistry assays. The current results warrant that further studies should be conducted to characterize the biological effects of these two polypeptides, to explore the potential for these peptides to be applied to the early screening of esophageal cancer, and to evaluate the potential for these peptides to mediate cell-targeted cancer therapies.

## Abbreviations

ELISA: Enzyme-linked immunosorbent assay; E. coli: Escherichia coli; FITC: Fluorescein isothiocyanate; LB-Tet: Luria-bertani-tetracycline; HRP: Horseradish peroxidase; LB: Luria broth; TMB: Tetramethylbenzidine; DAB: Diaminobenzidinetetrahydrochloride; PEG: Polyethylene glycol; TE: Tris-EDTA; Pfu: Plaque forming unit; TBST: Tris-buffered saline/Tween-20; NEE: Normal human esophageal epithelial cells; BSA: Bovine serum albumin; DNA: Deoxyribonucleic acid; Bp: Base pair.

## Competing interests

The authors declare that they have no competing interests.

## Authors’ contributions

YBC and ZFZ conceived and participated in the design of this study. ZFZ, XS, and YXW performed the experiments, completed the data analysis, interpretated the data, and drafted the manuscript. WW and SYF provided a draft of the manuscript as well as critical revisions. All authors have read and approved the final manuscript.
